# Development of MRI-Detectable Boron-Containing Gold Nanoparticle-Encapsulated Biodegradable Polymeric Matrix for Boron Neutron Capture Therapy (BNCT)

**DOI:** 10.3390/ijms22158050

**Published:** 2021-07-28

**Authors:** Chun-Yi Wu, Hsin-Hua Hsieh, Ting-Yu Chang, Jia-Jia Lin, Chin-Ching Wu, Ming-Hua Hsu, Ming-Chia Lin, Shin-Lei Peng

**Affiliations:** 1 Department of Biomedical Imaging and Radiological Sciences, National Yang Ming Chiao Tung University, Taipei 112, Taiwan; chunyiwu@ym.edu.tw (C.-Y.W.); alexa851024@gmail.com (H.-H.H.); tingyu870617@gmail.com (T.-Y.C.); 2 Department of Biomedical Imaging and Radiological Sciences, National Yang-Ming University, Taipei 112, Taiwan; 3 Department of Biomedical Imaging and Radiological Science, China Medical University, Taichung 404, Taiwan; solar3520@gmail.com; 4 Department of Public Health, China Medical University, Taichung 404, Taiwan; wucc@mail.cmu.edu.tw; 5 Department of Chemistry, National Changhua University of Education, Changhua 500, Taiwan; minghuahsu@cc.ncue.edu.tw; 6 Department of Nuclear Medicine, E-DA Hospital, Kaohsiung 824, Taiwan

**Keywords:** boron neutron capture therapy (BNCT), boron-containing nanoparticles, magnetic resonance imaging (MRI)

## Abstract

This study aimed to develop a novel magnetic resonance imaging (MRI)-detectable boron (B)-containing nanoassemblies and evaluate their potential for boron neutron capture therapy (BNCT). Starting from the citrate-coated gold nanoparticles (AuNPs) (23.9 ± 10.2 nm), the diameter of poly (D, L-lactide-co-glycolide) AuNPs (PLGA-AuNPs) increased approximately 110 nm after the encapsulation of the PLGA polymer. Among various B drugs, the self-produced B cages had the highest loading efficiency. The average diameter of gadolinium (Gd)- and B-loaded NPs (PLGA-Gd/B-AuNPs) was 160.6 ± 50.6 nm with a B encapsulation efficiency of 28.7 ± 2.3%. In vitro MR images showed that the signal intensity of PLGA-Gd/B-AuNPs in T1-weighted images was proportional to its Gd concentration, and there exists a significantly positive relationship between Gd and B concentrations (*R*^2^ = 0.74, *p* < 0.005). The hyperintensity of either 250 ± 50 mm^3^ (larger) or 100 ± 50 mm^3^ (smaller) N87 xenograft was clearly visualized at 1 h after intravenous injection of PLGA-Gd/B-AuNPs. However, PLGA-Gd/B-AuNPs stayed at the periphery of the larger xenograft while located near the center of the smaller one. The tumor-to-muscle ratios of B content, determined by inductively coupled plasma mass spectrometry, in smaller- and larger-sized tumors were 4.17 ± 1.42 and 1.99 ± 0.55, respectively. In summary, we successfully developed theranostic B- and Gd-containing AuNPs for BNCT in this study.

## 1. Introduction

Boron neutron capture therapy (BNCT), a cellular-level selective radiation therapy, is considered an attractive treatment strategy against cancers. Neither thermal/epithermal (low-energy) neutrons nor boron (B)-containing compounds alone can cause cell injury [1,2]. More specifically, the nuclear fission reaction only occurs when a ^10^B atom atom captures a neutron to produce a lithium-7 (^7^Li) recoil and an alpha particle, which would release their energy locally. Therefore, not all cells under the neutron-irradiated area are equally eradicated; only those already taking in non-radioactive ^10^B and then simultaneously receiving neutron irradiation are killed. Researchers worldwide devoted themselves to developing drugs or delivery systems to carry sufficient B atoms to malignant lesions rather than to healthy tissues.

The enhanced permeability and retention (EPR) effect [3] is based on leaky vasculature as well as poor lymphatic drainage in tumors; thus, nanoparticles (NPs) with diameters ranging from 100 to 200 nm can be selectively retained in the tumor. Poly(D, L-lactide-co-glycolide) (PLGA) is a Food and Drung Agency (FDA)-approved biodegradable and biocompatible polymer and has been extensively applied in the field of drug delivery because of its controlled release characteristics and low cytotoxicity. Even though many studies have revealed the potential of PLGA NPs to carry toxic payloads for cancer treatment [4], its feasibility for BNCT has not yet been widely investigated. Except for PLGA NPs, the feasibility of B-containing gold NPs (AuNPs) in BNCT has been summarized by Hosmane et al. [5,6,7]. Several groups have modified the surface of AuNPs with either carboranes or B cages through Click chemistry and reported promising results [8,9,10]. Previously, we also demonstrated the superior tumor targeting ability and tumor B content of anti-human epidermal receptor 2 (anti-HER2) antibody-modified B-containing AuNPs [11]. ^197^Au may be simultaneously activated by the neutron flux used for the activation of ^10^B atoms to form ^198^Au, which would release β-particles to cause tumor damage [12]. In addition, AuNPs can elevate oxidative stress and induce autophagic effect to damage tumor cells [13]. However, the total number of studies using AuNPs as carriers is not comparable with other drug delivery systems till now.

Except for ^10^B, gadolinium-157 (^157^Gd) (15.65% natural abundance), a stable (non-radioactive) atom with an extremely high cross section for thermal/epithermal neutrons, is also regarded as a potential element for neutron capture therapy (NCT). Gadolinium-based NCT (GdNCT) would produce internal conversion electrons and Auger electrons exert toxic effects on the cells where they are generated. Alberti et al. prepared Gd/B-containing folate-modified PLGA NPs for NCT to treat IGROV-1 ovarian cancer cells and demonstrated a superior in vitro killing effect [14]. Another attractive aspect of ^157^Gd is that Gd-based conjugates have been utilized as contrast agents to shorten the spin-lattice relaxation time for magnetic resonance imaging (MRI) studies. Tissues with higher Gd uptake would have better image contrast in T1-weighted (T1W) MR imaging. However, to the best of our knowledge, no study has determined the in vivo distribution of B- and Gd-containing PLGA NPs through MRI investigation as well as their therapeutic efficacy in NCT.

Extending from the previous BNCT studies, this study aimed to prepare the PLGA NPs encapsulating self-synthesized thiol B cage (BC-EG-SH), Gd atoms, and 20 nm AuNPs (PLGA-Gd/B-AuNPs) and to investigate their in vivo pharmacokinetics, and micro-distribution in the tumor.

## 2. Results

### 2.1. Physical Properties of NPs

**Figure 1:**
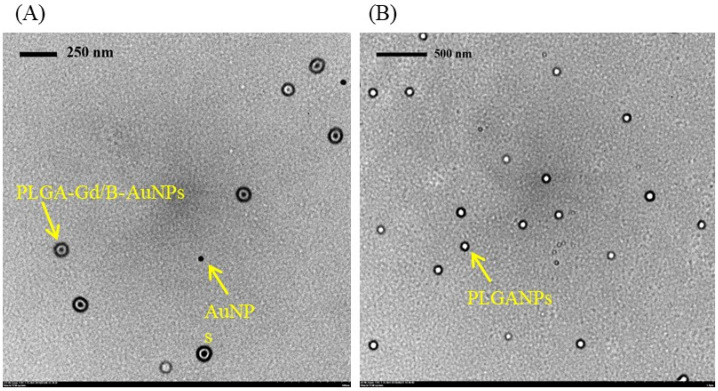
The TEM images of (**A**) PLGA-Gd/B-AuNPs and (**B**) PLGA NPs.

The diameter and B loading efficiency of various NPs are listed in Table 1. Starting from the commercial citrate-coated AuNPs (23.9 ± 10.2 nm), the diameter increased by approximately 110 nm after encapsulation into PLGA polymer (PLGA-AuNPs, 138.7 ± 29.1 nm). The particle size was similar to nude PLGA NPs (PLGA-NPs, 150.8 ± 38.6 nm). After loading the thiol B cage into PLGA-AuNPs to form PLGA-BC-AuNPs, its diameter slightly increased to 175.4 ± 41.68 nm with a low loading efficiency of 8.8 ± 3.2%. Enhanced sonication time did not obviously change the diameter of NPs but improved the loading efficiency of thiol B cage (reactions 4–6). However, similar results were not observed after adding either 4-borono-L-phenylalanine (boronophenylalanine, BPA) or BPA fructose complex (BPA–Fr) (reactions 7–12). In this study, based on the loading efficiency and size of NPs, the thiol B cage was selected as the B drug for encapsulation. For MRI-detectable NPs, the commercial Gd-based contrast was added into thiol B cage-containing PLGA-encapsulated AuNPs (reactions 13–15). As the longer sonication time and the two-fold amount of added thiol B cage did not significantly enhance the loading efficiency (reactions 13–15), the formulation of reaction 13 was selected for the following experiments. Transmission electron microscope (TEM) images demonstrated that PLGA polymers encapsulated the AuNPs despite some nude AuNPs existed (Figure 1). Interestingly, only AuNP alone was located in a single PLGA-encapsulated NP. No aggregated gold clusters were noticed in the NPs (Figure 1).

### 2.2. Assessment of In Vitro Cellular Uptake

**Figure 2:**
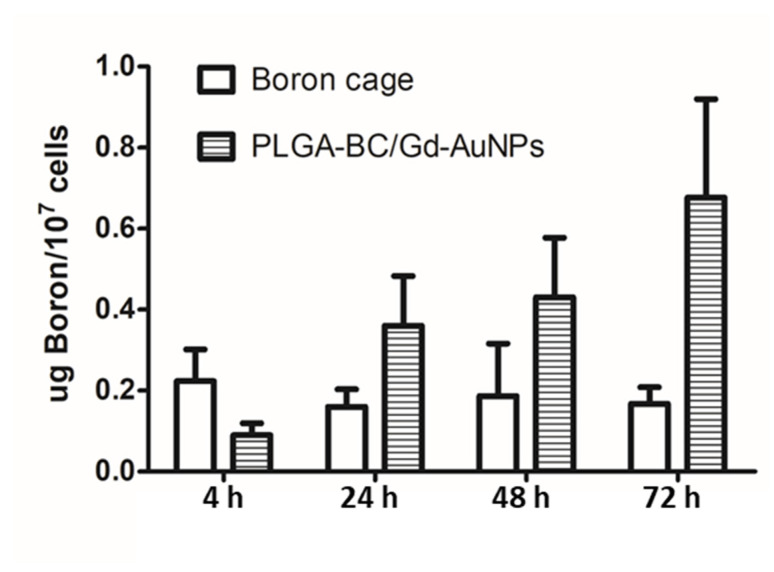
Cellular uptake of PLGA-Gd/B-AuNPs and unencapsulated thiol B cage in N87 cells at 4, 24, 48 and 72 h after incubation.

Cellular uptakes of PLGA-BC/Gd-AuNPs and thiol B cage were determined by the percentage of B atoms retained in cells (%AD/10^7^ cells). The accumulation of PLGA-BC/Gd-AuNPs in N87 cells increased with time, reaching a maximum of 0.68 ± 0.24 at 36-h post-incubation (Figure 2). However, the retention of thiol B cage was lower than that of PLGA-BC/Gd-AuNPs at almost all time points except for the initial 4 h (0.22 ± 0.24 for thiol B cage vs. 0.09 ± 0.03 for PLGA-BC/Gd-AuNPs), suggesting that PLGA encapsulation can improve the B content in N87 tumor cells.

### 2.3. In Vitro Phantom Imaging Studies

**Figure 3:**
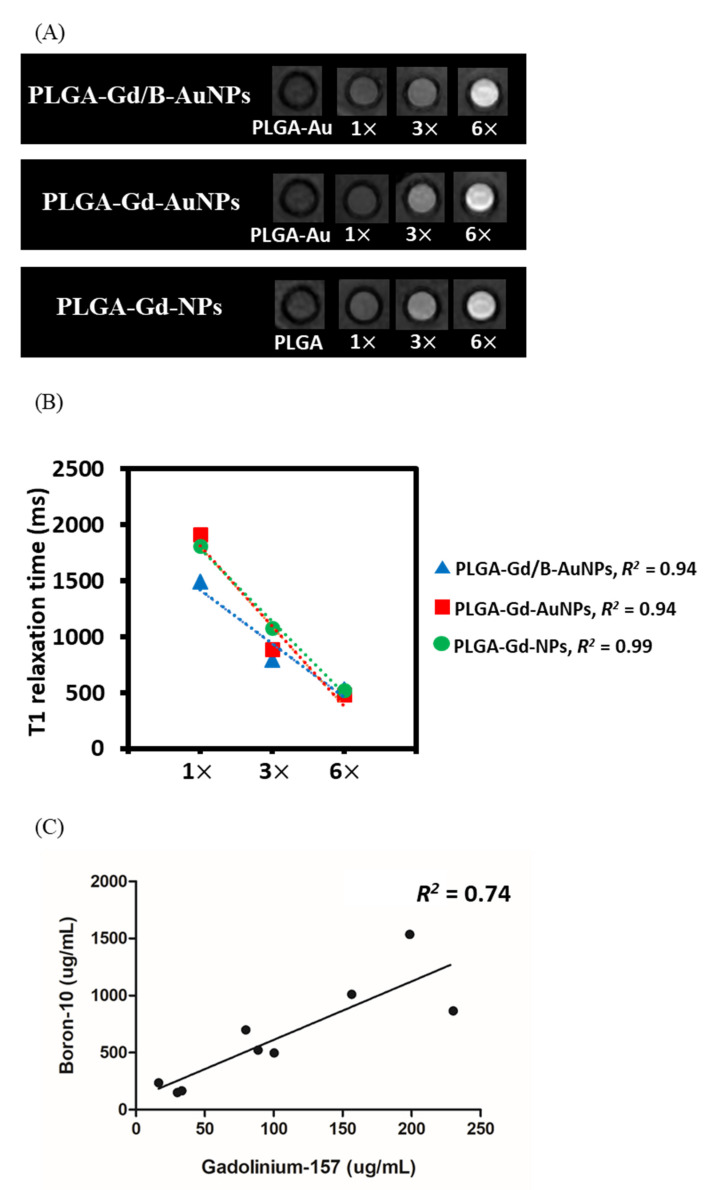
(**A**) T1-weighted MR images of nanoparticles at different Gd concentrations. (**B**) T1 relaxation time as a function of Gd concentration for different nanoparticles. (**C**) Correlation between B and Gd concentration obtained from inductively coupled plasma mass spectrometry. 1×: 26.6 µg/mL, 3×: 89.5 µg/mL, 6×: 195 µg/mL (gadolinium concentration).

T1W MR images of different concentrations of Gd in NPs are displayed in Figure 3A. As shown, the intensity of the signal increased for T1W images when the Gd concentration increased for PLGA-Gd/B-AuNPs, PLGA-Gd-AuNPs and PLGA-Gd-NPs. Conversely, no signal enhancement was observed in the control NPs (PLGA-AuNPs and PLGA-NPs). Significant reciprocal relationships were found between T1 relaxation and Gd concentration for all three Gd NPs, suggesting a concentration-dependent T1 shortening effect (Figure 3B). Moreover, regression analysis revealed a significantly positive correlation between B and Gd concentrations (Figure 3C, *R*^2^ = 0.74, *p* < 0.005). As the Gd concentration has a positive effect on MRI signals (Figure 3A), the tight relationship between B and Gd concentrations may imply that B concentration can be noninvasively evaluated by the MRI signal.

### 2.4. In Vivo MR Imaging of NPs in Tumor Xenografts

**Figure 4:**
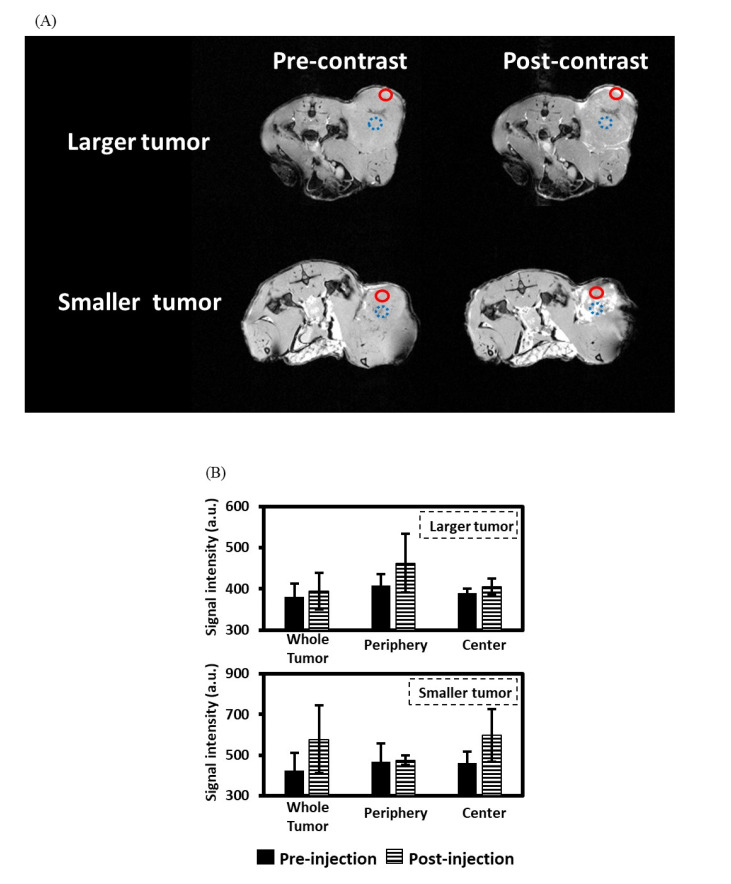
MR images from representative rats with either 250 ± 50 mm^3^ (larger) or 100 ± 50 mm^3^ (smaller) xenograft tumors. (**A**) MR images before and after 1 h PLGA-BC/Gd-AuNP injection. The red circles with a solid line indicate the location of the tumor periphery while the blue circles with a dashed line indicate the location of the tumor center. (**B**) Comparisons of signal intensities before and after 1 h PLGA-BC/Gd-AuNP injection.

T1W MR images of mice with N87 human gastric cancer cells in the flank before and after 1 h PLGA-Gd/B-AuNP injection are shown in Figure 4A. The hyperintensity of the tumor was clearly visualized in T1W MR images because of the T1-shortening effect caused by the NPs. The signal intensities of T1W MR images at different locations of the tumors are displayed in Figure 4B. The average signals of the whole tumor increased after NPs injection. More specifically, the PLGA-Gd/B-AuNPs are retained at the periphery of the 250 ± 50 mm^3^ (larger) xenograft while penetrating deeper into the 100 ± 50 mm^3^ (smaller) tumor.

### 2.5. Assessment of B Content in the Tumor and Muscle

B concentrations of tumor and muscle were determined by inductively coupled plasma mass spectrometry (ICP-MS). The intratumoral B concentrations of large- and small-sized xenograft-bearing mice were 24.25 ± 5.05 and 76.55 ± 11.75 µg/g sample, respectively, after animal MRI, while those of the tumor-to-muscle (T/M) ratios were 3.93 ± 5.05 and 1.84 ± 5.05, respectively (Table 2).

## 3. Discussion

To date, only BPA and sodium borocaptate (BSH) can be applied in clinical BNCT. Even though BSH has higher Bcontent, it requires a driving force for entering the tumor, resulting in a lower tumor-to-normal tissue ratio than BPA. PLGA, a FDA-approved material for human use, is made of lactide and glycolide and transformed to H_2_O and CO_2_ after hydrolysis without toxic metabolites. Moreover, PLGA has a pH-responsive property that can facilitate the release of B drugs within the tumor because of the acidic environment [15]. PLGA NPs have been considered useful carriers to deliver B compounds to the tumor [16,17,18]. However, the encapsulation efficiency of either o-carborane or B cage in the PLGA NPs was extremely low possibly due to their hydrophilic properties [16]. Takeuchi et al. developed o-carborane-loaded PLGA NPs with a diameter of 100–150 nm and demonstrated a beneficial effect on increasing B concentration in tumors [16]. They recently prepared chitosan-coated B-containing PLGA NPs and found that the B concentration of these NPs in B16 melanoma cells was 1.8-fold higher than that of bare ones (without chitosan modification) [17]. Shi et al. synthesized boronated porphyrin-loaded PLGA NPs (BPN) and noninvasively determined the B distribution by co-encapsulation with fluorescent dyes and copper-64 (^64^Cu) isotopes for optical and micro positron emission tomography (PET) imaging [18]. The T/M ratio of B16F10 melanoma xenograft-bearing mice that derived from micro PET, was greater than 50 at 24 h after five intravenous injections of BPN [18]. Therefore, the suppressed tumor volume was observed in the mice that received BPN plus neutron irradiation. In the present study, we found that the loading efficiency of the thiol B cage was significantly improved by the addition of AuNPs under identical conditions (Table 1). This may be due to the “concentrated” B cage caused by the thiol–Au linkage between the B cage and the surface of AuNPs [11]. Another evidence is that AuNPs cannot help with the B concentration of BPA- and BPA-Fr-loaded PLGA NPs because there is no bonding between these two drugs and AuNPs (Table 1). The improved solubility of BPA-fructose may account for the higher B content of BPA-fructose-loaded nanocomplexes when compared with that of BPA-loaded ones, especially in the water-in-oil-in-water preparation method [4]. We also found that the addition of Gd contrast (0.05 mmol) did not affect the size and the thiol B cage entrapped percentage. Moreover, the number of loaded Gd atoms, determined by ICP-MS, should be sufficient to track the pharmacokinetics of NPs by MRI (Table 1 and Figure 3).

The cellular uptake patterns of different carboranes or B cages are varied. Chou et al. reported that the accumulation of BSH in HepG2 cells is a time-dependent and reached 23 µg/g at 4 h post-incubation [19]. It is believed that the BSH uptake by the tumor cells relies mainly on passive diffusion. Therefore, Fukuo et al. conjugated a dodecaborate with a BPA molecule to form boronophenylalanine-amide alkyl dodecaborate (BADB) to achieve “active-targeting” [20]. However, the cellular uptake of BADA in F98 cells did not elevate with increasing incubation time, suggesting that the modification with BPA cannot improve the retention of dodecaborate. In contrast with the mechanism of small molecules transported into cells, NPs can be internalized to the cytoplasm via pinocytosis or phagocytosis [21,22]. In the present study, the B accumulation of PLGA-Gd/B-AuNPs in N87 cells increased with time even these B-containing NPs have not been modified with the active-targeting components, such as antibodies and peptides (Figure 2). By contrast, we did not observe the increasing cellular uptake of the thiol B cage in this study. B atoms required for successful BNCT is approximately 20–50 μg/g tumor or 10^9^ atoms/tumor cell [23]. Regarding the B uptake of PLGA-BC/Gd-AuNPs in N87 tumor cells that achieved 4.1 ×10^9^ atoms/tumor cell at 72 h after incubation, these nanocomplexes should have potential for future application. However, a similar phenomenon was not observed in B cage-treated cells, in contrast with the results reported by Chou et al. [19].

The success of BNCT depends on the specific delivery of sufficient ^10^B atoms to the tumor. Noninvasive imaging of the distribution of NPs in patients can help physicians quantify the tumor accumulation before treatment to exclude those who will not benefit from BNCT. Nakamura et al. and Takahashi et al. developed Gd-DTPA-conjugated carborane [24] and BPA [25] for MRI, respectively. Even though Gd-containing BPA has a higher tumor accumulation than Gd-containing carborane, this kind of conjugation would dramatically affect the pharmacokinetics of the original B drugs, suggesting that these conjugates did not serve as good surrogates of either carborane or BPA [24,25]. Geninatti-Crich et al. synthesized a hydrophobic dual Gd/B adduct that formed micelles in the aqueous solution and conjugated these micelles with β-cyclodextrin to provide amphiphilic complexes [26]. This group also revealed that these NPs can be tracked by MRI and lead to an effective tumor growth inhibition when the tumor-bearing mice received neutron irradiation [26]. Regarding the successful imaging ability and tumor-targeting efficiency of B- and Gd-containing NPs, the fractions of each atom (Au, B and Gd) in the nanocomplexes should be important. However, the number of AuNPs was added based on previous studies and the optimal fraction in the nanocomplexes was not determined in this pilot study. The ICP-MS analysis indicates that the B content of a small-sized tumor is approximately 70 μg/g tumor, which is higher than the clinical requirement of approximately 20 μg/g tumor. A promising treatment outcome can be expected when using enriched ^10^B-thiol B cage as a drug for encapsulation. For optimal Gd concentration in tumor for GdNCT, Deagostino et al. suggest that 50–200 μg/g tumor is needed [27]. In the present study, we did not determine the tumor Gd content because we treated Gd atoms as contrast agents rather than as therapeutics. MRI indicated the NPs were preferentially retained in the tumor; as a result, the T/M exceeded 2 in the mice bearing with either large- or small-sized tumors at 24 h p.i. due to the EPR effect, as expected (Figure 4). However, the localization patterns of nanoparticles in two tumor models were significantly different. The reason why the NPs were retained in the periphery of large-sized tumors may be attributed to tumor necrosis. Gaining knowledge of the “micro-distribution” of B drugs in the tumor may lead to a better understanding of radiation dosimetry and recurrence and highlight the notion that integrated diagnosis and therapy systems may offer benefits of personalized tailoring interventions. In this pilot study, we did not determine the pharmacokinetics of these NPs; thus, the maximum tumor accumulation and the most optimal neutron irradiation time point are still needed in further studies.

In addition to MRI contrast, ^157^Gd has a high cross section that can capture a thermal or epithermal neutron to form an unstable immediate and then undergoes gamma decay to produce internal conversion electrons and low-energy Auger electrons for cytotoxic effects. Alberti et al. indicated that ^157^Gd activation only contributed to a limited absorbed dose in NCT; thus, the additional killing effect of GdNCT is difficult to determine [28]. However, the application of Gd as an ideal theranostic agent for NCT has been proven by Deagostino et al. [27]. The present study further shows that MRI can provide more information about the micro-distribution of Gd atoms within the tumor, which may be beneficial to the dose escalation in GdNCT (Figure 4). In contrast with BNCT, gamma rays (approximately 2.2 MeV) can be produced in the GdNCT and raises the concern of distal healthy tissue damage. Fortunately, Le et al. found that the level of Gd accumulation is proportional to the tumor size and claimed that the radiation dose of healthy tissues would be not a problem. By contrast, it can be an additional advantage when treating a centimeters-sized tumor [29]. In addition, several recent studies have ignored the consequence of gamma rays on healthy cells [30,31,32], suggesting this should be of less importance in clinical therapy. The results of this study must be interpreted in view of some limitations. First, the additional killing effect of Gd is not determined. The additive effect of Gd in BNCT requires further verifications in the future. Second, only gastric cancer cells in the xenograft model was tested. An experiment similar to the current one but with a different cancer model is warranted.

## 4. Materials and Methods

### 4.1. Materials

**Figure 5:**
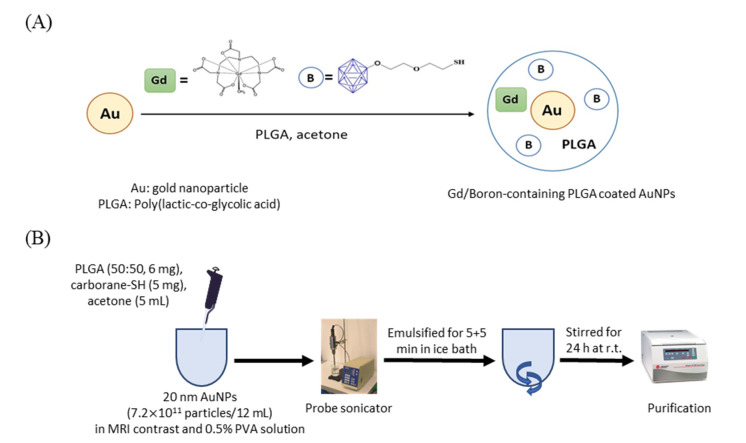
(**A**) Structure of self-synthesized thiol B cage (BC-EG-SH). (**B**) Synthetic scheme of PLGA nanoparticles.

Citrate-coated AuNPs (20 nm) were purchased from Sigma-Aldrich Co. (St. Louis, MO, USA). Gadopentetate dimeglumine (Magnevist^®^, BAYER, Leverkusen, Germany) was purchased from a local company. Thiol B cage-SH (BC-EG-SH, Figure 5A) was kindly provided by Prof. Ming-Hua Hsu. (4-Borono-L-phenylalanine (BPA) was purchased from Acros Organics Co. (#358932500, Fair Lawn, NJ, USA). The chemical purity of BPA is 97%. The B atoms in the thiol B cage and BPA were natural B, containing approximately 80% of ^11^B and 20% of ^10^B.) All other chemicals were purchased from Sigma-Aldrich Co. (St. Louis, MO, USA). Cell culture dishes, flasks and plasticware were purchased from Corning Inc (New York, NY, USA). Fetal bovine serum and culture medium were obtained from Thermo Fisher Scientific (Waltham, MA, USA).

### 4.2. Preparation of Gd/Au/B Cage-Loaded PLGA NPs (PLGA-Gd/B-AuNPs)

The scheme for the preparation of PLGA-Gd/B-AuNPs is shown in Figure 5B. For the preparation of BPA-Fr, 1 g of BPA was added to the flask containing fructose solution (2.2 g in 30 mL of ddH_2_O). Sodium hydroxide solution (1 N) was slowly added until the mixture became transparent (pH = 9.5–10.0). Finally, HCl (1 N) was used for neutralization (pH = 7.4) to give a BPA-Fr solution. Then, 6 mg of PLGA and 5 mg of B drugs (BC-EG-SH, BPA or BPA-Fr) were dissolved in 5 mL of acetone and dropwise added to 11 mL of 0.5% polyvinyl alcohol aqueous solution containing 0.05 mmol of gadopentetate dimeglumine and 1 mL of AuNP solution (7 × 10^11^ particles). The reaction mixture was emulsified by a probe sonicator (Branson Digital Sonifier 450, Emerson Electric Co., St. Louis, MO, USA) at 33 W of energy for 10 min in an ice bath. The emulsion was stirred at room temperature (r.t.) overnight. After the reaction, the mixture was centrifuged at 21,500 g (Himac CT15RE, Hitachi Koki Co., Tokyo, Japan) for 20 min. The resulting pellet was rinsed with ddH_2_O thrice to remove excess reagents. The controls (bareNPs) were synthesized with the identical protocol except for adding AuNPs (Table 1). The diameter of NPs was determined by the dynamic light scattering system (#ZS90, Malvern Panalytical Ltd., Malvern, UK). The morphology of NPs was assessed by TEM (HT7700, HITACHI, Ibaraki, Japan).

### 4.3. In Vitro Stability of NPs

Each of the PLGA NPs was incubated in either ddH_2_O at r.t. or in fetal bovine serum (FBS) at 37 °C for 0, 1, 2, 4 and 8 h. The percentage of NPs with the original size at each designated time point was considered an index of stability.

### 4.4. Assessment of Loading Efficiency of NPs

The B content of PLGA NPs loaded with BC-EG-SH, BPA or BPA-Fr was determined by ICP-MS (ELAN DRC ROMAN II ICP, Perkin-Elmer, Waltham, MA, USA). The protocol for ICP-MS sample preparation was based on the methods published in a previous study [11]. Briefly, the sample was digested with pure nitric acid (sample/HNO_3_ = 1/5, vol/vol) for 24 h at r.t. After homogenization, the diluted sample solution was used for the ICP-MS analysis. Blank samples (ddH_2_O and nitric acid) were prepared with an identical method for correction. The B standard used for the establishment of calibration curves was obtained from Sigma-Aldrich Co. (#0932, St. Louis, MO, USA).

### 4.5. Cell Culture and Xenograft Inoculation

N87 human gastric cancer cells were cultured in RPMI 1640 medium supplemented with 10% FBS at 37°C under 5% CO_2_ humidified atmosphere. For xenograft inoculation, N87 cells (1 × 10^6^) in 100 μL of Matrigel and serum-free medium mixture (1:1, vol/vol) were subcutaneously implanted into the right flank of a 6-week-old male mouse with severe combined immunodeficiency. The animals were purchased from the National Laboratory Animal Center, Taiwan. The tumor-bearing mice were randomly divided into two groups. The mice in the two groups were subjected to further experiments when the tumor size reached 100 ± 50 mm^3^ and 250 ± 50 mm^3^, respectively.

### 4.6. In Vitro Cellular Uptake Assays

Approximately 5 × 10^6^ cells were seeded in a 10 cm dish and cultured in culture medium containing 10% FBS (10 mL) overnight. When cells reached 70% confluency, the culture medium was replaced by the NP-containing serum-free medium (1 mg B atoms for each group). At 0.5, 1, 2 and 4 h post-incubation, the medium was aspirated and cells were washed twice with PBS (0.5 mL) to remove unbound NPs. Cells were treated with 0.5 mL of 0.25% trypsin for 5 min for detachment from the plate. The cells were then collected, and the number of cells in the cell suspension was measured using a hemocytometer for normalization of cellular uptake values in each group. The B concentration in cells was determined by ICP-MS. The cellular uptake of NPs was expressed as the B concentration in cells (µg B/10^7^ cells).

### 4.7. Phantom Imaging

Each of the NP-containing solutions (PLGA-Gd/B-AuNPs, PLGA-Gd-AuNPs, PLGA-Gd-NPs, PLGANPs and PLGA-AuNPs) was centrifuged and concentrated to produce 1-, 3- and 6-strength (1×, 3× and 6×) concentrates (in 1 mL H_2_O). The Gd concentration for 1×, 3× and 6× were 26.6, 89.5, ad 195 µg/mL, respectively, measured by ICP-MS. To verify the ability of PLGA NPs as MRI contrast agent, MR-T1 relaxation times of these nanoparticles were determined using a MRI scanner (SIGNA HDxt, GE, Milwaukee, WI, USA). The pulse sequences of inversion recovery were as follows: repetition time (TR) = 15,000 ms, echo time (TE) = 16.94 ms, inversion time (TI) = 50–800 ms (50 ms intervals), slice thickness = 3 mm, matrix size = 512 × 512 and field of view (FOV) = 118 mm × 118 mm. The images were analyzed using custom Matlab scripts [33]. Spin-lattice T1 relaxation was calculated as follows: M = M_0_ ×(1-e^−TI/T1^), where M_0_ is the magnetic moment at equilibrium. In addition, B and Gd concentrations of another 6 PLGA-Gd/B-AuNPs samples were analyzed by the ICP-MS. Therefore, a total of nine samples were used to determine the relationship between B and Gd concentrations.

### 4.8. Animal MRI

The animal MRI experiments were conducted using a 7T animal MRI scanner (Bruker ClinScan 70/30, Billerica, MA, USA). Mice were anesthetized with 4% isoflurane, which as reduced to 2.5% isoflurane for maintenance. A 30-gauge needle connected to a 0.8 m polyethylene-30 tube was inserted into the tail vein for the injection of PLGA-Gd/B-AuNPs (3 mg B/mL, 500 µL). A T1W fast low angle shot was used before and after 1 h sample injection with the following parameters: TR = 300 ms, TE = 3.81 ms, slice thickness = 0.8 mm, matrix size = 256 × 160 and FOV = 37 mm × 60 mm.

### 4.9. Assessment of B Content in Tumor and Muscle

The mice in each group (*n* = 2) were sacrificed at 24 h p.i. The N87 xenograft and muscle were excised, weighed and homogenized for ICP-MS analysis (ELAN DRC ROMAN II, Perkin-Elmer, Waltham, MA, USA). The B contents were expressed as μg/g tissue.

### 4.10. Statistical Analysis

All values were expressed as mean ± standard derivation. The Student’s *t*-test was used for the comparison between groups. The relationship between B and Gd concentrations from the nine PLGA-Gd/B-AuNPs samples was assessed by Pearson correlation coefficient analysis. Values of *p* < 0.05 were regarded as significant.

## 5. Conclusions

In this study, we successfully developed MRI-detectable B- and Gd-containing PLGA NPs and found that self-synthesized thiol B cage (BC-EG-SH) is the most optimal B compound for encapsulation among three different molecules as its encapsulation efficiency could be enhanced by the addition of AuNPs, reaching approximately 30%. Moreover, the diagnostic ability of Gd was not affected by the PLGA-loaded AuNPs, paving the way for future studies regarding the noninvasive imaging of the distribution of NPs in patients. These PLGA NPs have beneficial effects on providing a delivery system for anticancer treatments and an alternative diagnostic technique that can facilitate future clinical trials.

## Figures and Tables

**Table 1:** The formulation and physical properties of various nanoparticles.

		*AuNPs (Particles)	B Drug	Gd Contrast (mmol)	PLGA/PVA	Sonication (min)	Diameter (nm)	Loading Efficiency (%)
1.	AuNPs	7 × 10^11^	---	---	---	---	23.9 ± 10.2	---
2.	PLGA-NPs	---	---	---	6 mg/11 mL of 0.5% PVA solution	10	150.8 ± 38.6	---
3.	PLGA-AuNPs	7 × 10^11^	---	---	6 mg/11 mL of 0.5% PVA solution	10	138.7 ± 29.1	---
4.	PLGA-BC	---	BC-EG-SH; 5 mg	---	6 mg/11 mL of 0.5% PVA solution	10	202.3 ± 78.1	7.3 ± 1.7
5.	PLGA-BC-AuNPs	7 × 10^11^	BC-EG-SH; 5 mg	---	6 mg/11 mL of 0.5% PVA solution	5	180.1 ± 53.3	8.6 ± 1.7
6.	PLGA-BC-AuNPs	7 × 10^11^	BC-EG-SH; 5 mg	---	6 mg/11 mL of 0.5% PVA solution	10	175.4 ± 41.7	29.0 ± 5.1
7.	PLGA-BC-AuNPs	7 × 10^11^	BC-EG-SH; 5 mg	---	6 mg/11 mL of 0.5% PVA solution	20	169.2 ± 33.5	35.4 ± 5.0
8.	PLGA-BPA-AuNPs	7 × 10^11^	BPA; 5 mg	---	6 mg/11 mL of 0.5% PVA solution	5	395.4 ± 101.9	3.1 ± 2.5
9.	PLGA-BPA-AuNPs	7 × 10^11^	BPA; 5 mg	---	6 mg/11 mL of 0.5% PVA solution	10	374.2 ± 90.7	2.7 ± 1.9
10.	PLGA-BPA-AuNPs	7 × 10^11^	BPA; 5 mg	---	6 mg/11 mL of 0.5% PVA solution	20	366.2 ± 72.8	3.0 ± 1.5
11.	PLGA-BPAFr-AuNPs	7 × 10^11^	BPA-Fr; 5 mg	---	6 mg/11 mL of 0.5% PVA solution	5	215.9 ± 40.1	9.2 ± 5.8
12.	PLGA-BPAFr-AuNPs	7 × 10^11^	BPA-Fr; 5 mg	---	6 mg/11 mL of 0.5% PVA solution	10	197.7 ± 33.2	8.7 ± 3.2
13.	PLGA-BPAFr-AuNPs	7 × 10^11^	BPA-Fr; 5 mg	---	6 mg/11 mL of 0.5% PVA solution	20	188.2 ± 31.1	9.0 ± 2.7
14.	PLGA-BC/Gd-AuNPs	7 × 10^11^	BC-EG-SH; 5 mg	0.05	6 mg/11 mL of 0.5% PVA solution	10	160.6 ± 50.6	28.7 ± 2.3
15.	PLGA-BC/Gd-AuNPs	7 × 10^11^	BC-EG-SH; 5 mg	0.05	6 mg/11 mL of 0.5% PVA solution	20	158.3 ± 45.4	29.0 ± 8.0
16.	PLGA-BC/Gd-AuNPs	7 × 10^11^	BC-EG-SH; 10 mg	0.05	6 mg/11 mL of 0.5% PVA solution	10	172.8 ± 68.5	31.1 ± 2.5
17.	PLGA-BC/Gd-NPs	---	BC-EG-SH; 5 mg	0.05	6 mg/11 mL of 0.5% PVA solution	10	176.4 ± 59.3	19.1 ± 7.9

* The amount of added AuNPs was calculated based on the concentration of commercial AuNP solution (7 × 10^11^ particles/mL) as one milliliter of the solution was used for the preparation.

**Table 2:** Boron content in tumor and muscle determined by inductively coupled plasma mass spectrometry.

	Muscle (µg /g)	Tumor (µg /g)	T/M *
Small-sized	16.73 ± 4.66	69.73 ± 13.60	4.29 ± 0.51
Large-sized	12.03 ± 2.36	23.93 ± 4.62	2.12 ± 0.75

* T/M: tumor-to-muscle ratio.

## Data Availability

The data can be freely given upon request.

## References

[1] Barth R.F., Zhang Z., Liu T. (2018). A realistic appraisal of boron neutron capture therapy as a cancer treatment modality. Cancer Commun..

[2] Sauerwein W., Wittig A., Moss R., Nakagawa Y. (2012). Neutron Capture Therapy. Principles and Applications.

[3] Maeda H., Wu J., Sawa T., Matsumura Y., Hori K. (2000). Tumor vascular permeability and the EPR effect in macromolecular therapeutics: A review. J. Control. Release.

[4] Rezvantalab S., Drude N.I., Moraveji M.K., Guvener N., Koons E.K., Shi Y., Lammers T., Kiessling F. (2018). PLGA-Based Nanoparticles in Cancer Treatment. Front. Pharmacol..

[5] Hosmane N.S. (2016). Boron Science: New Technologies and Applications.

[6] Hosmane N.S. (2019). Handbook of Boron Science.

[7] Hosmane N.S. (2012). Boron and Gadolinium Neutron Capture Therapy for Cancer Treatment.

[8] Cioran A.M., Teixidor F., Krpetic Z., Brust M., Vinas C. (2014). Preparation and characterization of Au nanoparticles capped with mercaptocarboranyl clusters. Dalton Trans..

[9] Li N., Zhao P., Salmon L., Ruiz J., Zabawa M., Hosmane N.S., Astruc D. (2013). “Click” star-shaped and dendritic PEGylated gold nanoparticle-carborane assemblies. Inorg. Chem..

[10] Liang L.Y., Rapakousiou A., Salmon L.R.J., Astruc D., Dash B.P., Satapathy R., Sawicki J.W. (2011). Assembly of Carborane-Appended Polymers and Stabilization of Gold and Palladium Nanoparticles. Eur. J. Inorg. Chem..

[11] Wu C.Y., Lin J.J., Chang W.Y., Hsieh C.Y., Wu C.C., Chen H.S., Hsu H.J., Yang A.S., Hsu M.H., Kuo W.Y. (2019). Development of theranostic active-targeting boron-containing gold nanoparticles for boron neutron capture therapy (BNCT). Colloids Surf. B Biointerfaces.

[12] Xuan S., de Barros A., Nunes R.C., Ricci-Junior E., da Silva A.X., Sahid M., Alencar L.M.R., Dos Santos C.C., Morandi V., Alexis F. (2020). Radioactive gold nanocluster (198-AuNCs) showed inhibitory effects on cancer cells lines. Artif. Cells Nanomed. Biotechnol..

[13] Kubota T., Kuroda S., Kanaya N., Morihiro T., Aoyama K., Kakiuchi Y., Kikuchi S., Nishizaki M., Kagawa S., Tazawa H. (2018). HER2-targeted gold nanoparticles potentially overcome resistance to trastuzumab in gastric cancer. Nanomedicine.

[14] Alberti D., Protti N., Franck M., Stefania R., Bortolussi S., Altieri S., Deagostino A., Aime S., Geninatti Crich S. (2017). Theranostic Nanoparticles Loaded with Imaging Probes and Rubrocurcumin for Combined Cancer Therapy by Folate Receptor Targeting. ChemMedChem.

[15] Khanal S., Adhikari U., Rijal N.P., Bhattarai S.R., Sankar J., Bhattarai N. (2016). pH-Responsive PLGA Nanoparticle for Controlled Payload Delivery of Diclofenac Sodium. J. Funct. Biomater..

[16] Takeuchi I., Nomura K., Makino K. (2017). Hydrophobic boron compound-loaded poly(l-lactide-co-glycolide) nanoparticles for boron neutron capture therapy. Colloids Surf. B Biointerfaces.

[17] Takeuchi I., Ariyama M., Makino K. (2019). Chitosan Coating Effect on Cellular Uptake of PLGA Nanoparticles for Boron Neutron Capture Therapy. J. Oleo Sci.

[18] Shi Y., Li J., Zhang Z., Duan D., Zhang Z., Liu H., Liu T., Liu Z. (2018). Tracing Boron with Fluorescence and Positron Emission Tomography Imaging of Boronated Porphyrin Nanocomplex for Imaging-Guided Boron Neutron Capture Therapy. ACS Appl. Mater. Interfaces.

[19] Chou F.I., Chung H.P., Liu H.M., Chi C.W., Lui W.Y. (2009). Suitability of boron carriers for BNCT: Accumulation of boron in malignant and normal liver cells after treatment with BPA, BSH and BA. Appl. Radiat. Isot..

[20] Fukuo Y., Hattori Y., Kawabata S., Kashiwagi H., Kanemitsu T., Takeuchi K., Futamura G., Hiramatsu R., Watanabe T., Hu N. (2020). The Therapeutic Effects of Dodecaborate Containing Boronophenylalanine for Boron Neutron Capture Therapy in a Rat Brain Tumor Model. Biology.

[21] Hillaireau H., Couvreur P. (2009). Nanocarriers’ entry into the cell: Relevance to drug delivery. Cell Mol. Life Sci..

[22] Xie X., Liao J., Shao X., Li Q., Lin Y. (2017). The Effect of shape on Cellular Uptake of Gold Nanoparticles in the forms of Stars, Rods, and Triangles. Sci. Rep..

[23] Barth R.F., Mi P., Yang W. (2018). Boron delivery agents for neutron capture therapy of cancer. Cancer Commun..

[24] Nakamura H., Fukuda H., Girald F., Kobayashi T., Hiratsuka J., Akaizawa T., Nemoto H., Cai J., Yoshida K., Yamamoto Y. (2000). In vivo evaluation of carborane gadolinium-DTPA complex as an MR imaging boron carrier. Chem. Pharm. Bull..

[25] Takahashi K., Nakamura H., Furumoto S., Yamamoto K., Fukuda H., Matsumura A., Yamamoto Y. (2005). Synthesis and in vivo biodistribution of BPA-Gd-DTPA complex as a potential MRI contrast carrier for neutron capture therapy. Bioorg Med. Chem..

[26] Geninatti-Crich S., Alberti D., Szabo I., Deagostino A., Toppino A., Barge A., Ballarini F., Bortolussi S., Bruschi P., Protti N. (2011). MRI-guided neutron capture therapy by use of a dual gadolinium/boron agent targeted at tumour cells through upregulated low-density lipoprotein transporters. Chemistry.

[27] Deagostino A., Protti N., Alberti D., Boggio P., Bortolussi S., Altieri S., Crich S.G. (2016). Insights into the use of gadolinium and gadolinium/boron-based agents in imaging-guided neutron capture therapy applications. Future Med. Chem..

[28] Alberti D., Protti N., Toppino A., Deagostino A., Lanzardo S., Bortolussi S., Altieri S., Voena C., Chiarle R., Geninatti Crich S. (2015). A theranostic approach based on the use of a dual boron/Gd agent to improve the efficacy of Boron Neutron Capture Therapy in the lung cancer treatment. Nanomedicine.

[29] Le U.M., Cui Z. (2006). Biodistribution and tumor-accumulation of gadolinium (Gd) encapsulated in long-circulating liposomes in tumor-bearing mice for potential neutron capture therapy. Int. J. Pharm..

[30] Jung K.H., Park J.A., Kim J.Y., Kim M.H., Oh S., Kim H.K., Choi E.J., Kim H.J., Do S.H., Lee K.C. (2018). Image-Guided Neutron Capture Therapy Using the Gd-DO3A-BTA Complex as a New Combinatorial Treatment Approach. Contrast Media Mol. Imaging.

[31] Dewi N., Mi P., Yanagie H., Sakurai Y., Morishita Y., Yanagawa M., Nakagawa T., Shinohara A., Matsukawa T., Yokoyama K. (2016). In vivo evaluation of neutron capture therapy effectivity using calcium phosphate-based nanoparticles as Gd-DTPA delivery agent. J. Cancer Res. Clin. Oncol..

[32] Dewi N., Yanagie H., Zhu H., Demachi K., Shinohara A., Yokoyama K., Sekino M., Sakurai Y., Morishita Y., Iyomoto N. (2013). Tumor growth suppression by gadolinium-neutron capture therapy using gadolinium-entrapped liposome as gadolinium delivery agent. Biomed. Pharmacother..

[33] MathWorks, Inc. (2020). Matlab; Version R2020a.

